# Control of foreign polypeptide localization in specific layers of protein body type I in rice seed

**DOI:** 10.1007/s00299-016-1960-8

**Published:** 2016-02-24

**Authors:** Ai Sasou, Takanari Shigemitsu, Yuhi Saito, Manami Tanaka, Shigeto Morita, Takehiro Masumura

**Affiliations:** Laboratory of Genetic Engineering, Graduate School of Life and Environmental Sciences, Kyoto Prefectural University, Shimogamo, Kyoto, 606-8522 Japan; Biotechnology Research Department, Kyoto Prefectural Agriculture, Forestry, and Fisheries Technology Research Center, Kitainayazuma, Seika-cho, Soraku-gun, Kyoto, 619-0244 Japan

**Keywords:** Rice, Seed storage protein, Protein body type I, Prolamin, Green fluorescent protein

## Abstract

*****Key message***:**

**Prolamin–GFP fusion proteins, expressed under the control of native prolamin promoters, were localized in specific layers of PB-Is. Prolamin–GFP fusion proteins were gradually digested from outside by pepsin digestion.**

**Abstract:**

In rice seed endosperm, protein body type I (PB-I) has a layered structure consisting of prolamin species and is the resistant to digestive juices in the intestinal tract. We propose the utilization of PB-Is as an oral vaccine carrier to induce mucosal immune response effectively. If vaccine antigens are localized in a specific layer within PB-Is, they could be protected from gastric juice and be delivered intact to the small intestine. We observed the localization of GFP fluorescence in transgenic rice endosperm expressing prolamin–GFP fusion proteins with native prolamin promoters, and we confirmed that the foreign proteins were located in specific layers of PB-Is artificially. Each prolamin–GFP fusion protein was localized in specific layers of PB-Is, such as the outer-most layer, middle layer, and core region. Furthermore, to investigate the resistance of prolamin–GFP fusion proteins against pepsin digestion, we performed in vitro pepsin treatment. Prolamin–GFP fusion proteins were gradually digested from the peripheral region and the contours of PB-Is were made rough by in vitro pepsin treatment. These findings suggested that prolamin–GFP fusion proteins accumulating specific layers of PB-Is were gradually digested and exposed from the outside by pepsin digestion.

## Introduction

Rice is one of the world’s most important food crops. The major component of rice is carbohydrate, but it also contains seed storage proteins that are essential nutrition for humans (Shewry and Halford 2002). Rice seed endosperm accumulates storage proteins as a nitrogen source for germination and the early growth of the next generation. Rice seed storage proteins were categorized as alcohol-soluble prolamins, dilute acid/alkali-soluble glutelins, and saline-soluble globulins (Shewry et al. 1995). These storage proteins accumulate in protein body type I (PB-I) and protein body type II (PB-II) (Tanaka et al. 1980). PB-I is derived from endoplasmic reticulum (ER), and prolamins accumulate in PB-I. PB-II is a protein storage vacuole, and glutelins and globulins accumulate in PB-II. Prolamins are synthesized on rough ER and then accumulate into ER (Yamagata et al. 1982; Yamagata and Tanaka 1986). Prolamins are classified according to their molecular sizes: 16, 13 and 10 kDa. Furthermore, 13 kDa prolamins are divided into 13a-1, 13a-2, 13b-1, and 13b-2 prolamins (Saito et al. 2012), in which 13a-1 and 13a-2 prolamins possess Cys residues (Cys-rich type), but 13b-1 and 13b-2 prolamins possess no Cys residues in the mature form (Cys-less type). Prolamins together consist of a multigene family including 34 prolamin genes in the rice genome. There are 2, 2, 4, 4, 18, and 4 genes for 16, 13a-1, 13a-2, 13b-1, 13b-2, and 10 kDa prolamins, respectively (Saito et al. 2012).

In our previous study, the expression of 10 kDa prolamin mRNA began to increase first and then the expression of 13b-1 prolamin mRNA began to increase in slightly faster than that of 13a-1, 13a-2 and 16 kDa prolamin mRNAs. And the expression of 13b-2 prolamin mRNA occurred after the expression of the other prolamin mRNAs in the developing rice seeds. In response to temporal expression of each prolamin gene, 10 kDa prolamins were localized in the core region of PB-Is, 13b-1 prolamins were in the outer layer surrounding the 10 kDa prolamin-rich core, 13a (13a-1 and 13a-2) prolamins and 16 kDa prolamins were accumulated in the middle layer of PB-Is, and 13b-2 prolamins were in the outer-most layer (Saito et al. 2012). 13a prolamin–GFP fusion proteins expressed under the control of CaMV35S promoter accumulated in the core region of PB-Is (Saito et al. 2009). Furthermore, 13a prolamin–GFP fusion protein and 10 kDa prolamin–GFP fusion protein, both of which are expressed under the control of each native prolamin promoter, accumulated in the same region as each native prolamin’s localization (Saito et al. 2012). Thus, each prolamin promoter plays an important role in the localization of each prolamin polypeptide in the PB-Is.

Recently, plants have become attractive candidates for the production of beneficial foreign polypeptides. Since the beginning of the 1990s, many researchers have been studying the expression of vaccine antigens in transgenic plants as vaccine antigen hosts (Yusibov et al. 2011). For example, some antigen peptides and antibodies are produced using the transient expression system in tobacco leaves (Hamorsky et al. 2013). Matsui et al. (2009, 2011) demonstrated that lettuce is suitable for vaccine production because lettuce can be cultivated in a closed system under tightly controlled conditions for pharmaceutical manufacturing. Some researchers tried to develop a transgenic potato containing large amounts of proteins as a plant-based vaccine (Arakawa et al. 1998; Kim et al. 2004; Choi et al. 2005). Tomato is also utilized for pharmaceutical production because it can be eaten raw (Jiang et al. 2007; He et al. 2008; Sharma et al. 2008). As described above, although plant-based vaccines have the potential to produce pharmaceutical polypeptides, in practice there are some problems to overcome. When a plant-based oral vaccine is administered, the vaccine antigens are digested in the gastrointestinal tract, so a large amount of plant biomass must be administered. We propose that PB-Is in rice seeds are the most attractive candidate for the development of a plant-based vaccine as rice seed PB-Is are not digested in the human or rat gastrointestinal tract (Tanaka et al. 1975; Kubota et al. 2010, 2014). So Nochi et al. (2007) generated a rice-based edible vaccine that produces cholera toxin B subunit (CTB) and showed that CTB accumulated in rice seed PB-Is mainly induced a mucosal immune response.

Thus, we tried to develop PB-Is in rice seeds to carry and deliver vaccines. If a vaccine antigen can be localized in a specific layer of PB-Is, it can reach the gastrointestinal tracts without being digested by gastric acid or protease enzymes. Furthermore, such an antigen is expected to induce a mucosal immune response effectively at a low dose. To develop PB-Is as oral vaccine carriers, we tried to establish control over foreign protein localization within PB-Is. In this study, we generated transgenic rice expressing 13b-2 prolamin–GFP fusion protein under the control of the native promoter (13b-2P-GFP), in addition to other two transgenic rice lines (13a-1P-GFP and 10kP-GFP) used in a previous report (Saito et al. 2012). Furthermore, we assessed the relationship between GFP localization within PB-Is and digestibility of pepsin by in vitro pepsin treatment.

## Materials and methods

### Construction

**Fig. 1:**
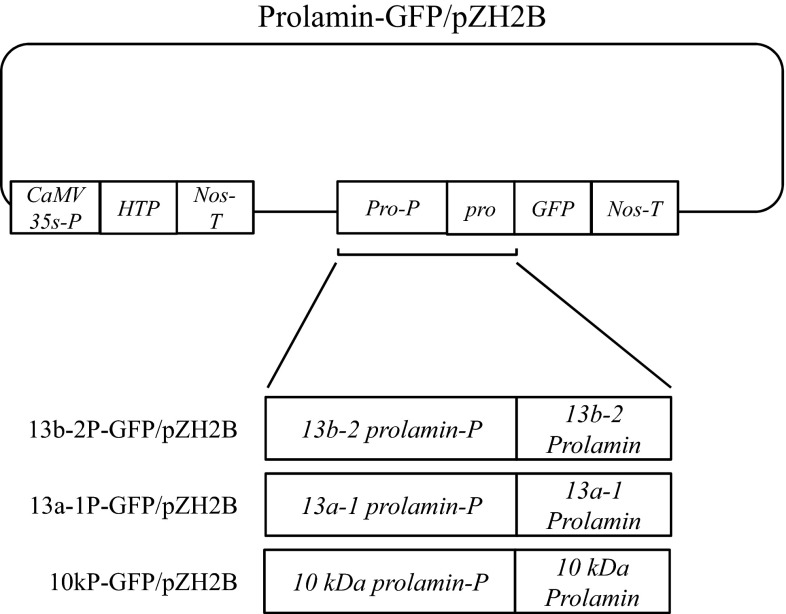
Constructs of prolamin–GFP that were expressed in the transgenic rice seeds. *CaMV35S-P* cauliflower mosaic virus 35S promoter, *HTP* hygromycin phosphotransferase, *Nos-T* nopaline synthase terminator, *Pro-P* prolamin promoter, *Pro* prolamin, *GFP* green fluorescent protein, *13b-2 prolamin-P* 13b-2 prolamin promoter, *13a-1 prolamin-P* 13a-1 prolamin promoter, *10* *kDa prolamin-P* 10 kDa prolamin promoter, *13b-2 prolamin* the coding sequence for the 13b-2 prolamin gene (λRM4, Os05g0329300), *13a-1 prolamin* the coding sequence for 13a-1 prolamin (λRM1, Os07g0206500), *10* *kDa prolamin* the coding sequence for 10 kDa prolamin (λRP10, Os03g0766100)

The constructs containing the cassettes of the coding regions of prolamin–GFP fusion proteins were generated based on pZH2B (Kuroda et al. 2010). The construction of 13a-1P-GFP and that of 10kP-GFP was described previously (Saito et al. 2012). The promoter and coding region of 13b-2 prolamin (Os05g0329300) was amplified from genome DNA of young rice leaves as templates using the forward primer (5′-gtcgactgtccatcattcctaacaagagg-3′ containing *Sal*I site) and reverse primer (5′-ttggatcccaagacaccgccaagggtgg-3′ containing *Bam*HI). The PCR fragment of the 13b-2 prolamin promoter and coding region was digested with *Sal*I and *Bam*HI, and inserted upstream of the GFP-NOS cassette of the vector of 13a-1P-GFP (Fig. 1). The resulting binary vectors were introduced into rice calli using an *Agrobacterium*-mediated method (Hiei et al. 1994). The rice calli containing the transgene were selected by hygromycin B (Nacalai Tesque, Kyoto, Japan) and transferred to redifferentiation medium. Then, the shoots from selected calli were transferred to plant pots containing soil.

### Plant material and growth conditions

The transgenic rice plants were grown in the plant pots in a naturally illuminated temperature-controlled (28 °C) greenhouse at the Biotechnology Research Department, Kyoto Prefectural Agriculture, Forestry, and Fisheries Technology Research Center, Japan.

### Protein analysis

For the extraction of total protein, the flour of mature seeds (10 mg) was homogenized in SDS sample buffer [62.5 mM Tris–HCl (pH 6.8), 4 M urea, 2 % (w/v) SDS, 0.1 M dithiothreitol] for 1 h. The homogenates were centrifuged at 15,000*g* for 5 min to obtain the protein extracts as supernatant solutions. The extracts were heated at 100 °C for 5 min. The RC DC Protein Assay Kit (Bio-Rad, Hercules, CA, USA) was used to measure the total proteins according to the manufacturer’s instructions. The 10 μg aliquots of proteins were analyzed by SDS-PAGE and immunoblotting. For SDS-PAGE analysis, the polypeptide bands were visualized by Coomassie brilliant blue R-250 staining. The separated polypeptides after SDS-PAGE were electrotransferred to an Immun-Blot PVDF Membrane (Bio-Rad), revealed using anti-GFP antibody (dilution 1:5000; Medical & Biological Laboratories, Nagoya, Japan), and reacted with the alkaline phosphatase (AP)-conjugated goat anti-rabbit IgG secondary antibody (1:20,000; Promega, Madison, WI, USA). AP-labeled bands were detected with 5-bromo-4-chloro-3-indoyl phosphate and nitroblue tetrazolium by using BCIP/NBT Color Development Substrate (Promega) according to the manufacturer’s instructions. Then each lane of the blot was scanned using Kodak 1D Image Analysis Software (Eastman Kodak, Rochester, NY, USA) to determine the staining intensities of individual polypeptides. The accumulation levels of prolamin–GFP fusion proteins in transgenic rice seeds were calibrated to known concentrations of recombinant GFP (Roche, Basal, Switzerland). Finally, 1, 3, 10, and 30 ng of recombinant GFP were loaded as calibration curves for immunoblot analysis.

### Fluorescence microscopic analysis

Thin sections of mature seeds were prepared in accordance with the frozen film method described by Saito et al. (2008). The prepared thin sections (2 μm) were stained with 10 nM rhodamine B, which is a pigment that stains the peripheral region of PB-I, for 10 min. Stained sections were observed with a fluorescence microscope (BX51; Olympus, Tokyo, Japan), and the images were analyzed with an Aquacosmos system (Hamamatsu Photonics, Hamamatsu, Japan).

### In vitro pepsin digestion

In vitro digestion analysis was a modified version of the method of Kumagai et al. (2006). The flour of mature seeds (100 mg) was incubated with 0.2 mg/mL pepsin (Sigma-Aldrich, St. Louis, MO, USA) in 5 mL of 0.01 M HCl buffer at 37 °C. To assess temporal changes, samples were taken up at 2, 4, 5, 6, 7, and 8 h from the start of pepsin digestion. After neutralization using 0.1 M NaOH, each sample was centrifuged at 15,000*g* for 5 min. The total proteins after the pepsin digestion were extracted from the pellets and analyzed by immunoblot analysis. After bands were detected, the staining intensities of individual bands were calculated by Kodak 1D Image Analysis Software (Eastman Kodak). Then, the ratio of undigested prolamin–GFP fusion proteins at each treated time was estimated by using the intensity values at 0 h treated samples as the criteria.

### Immuno-transmission electron microscopy

The pellets after pepsin digestion were used for immuno-transmission electron microscopic observation. The sample preparation was conducted using the methods described by Kubota et al. (2010). The pellets were suspended in 1 % (w/v) agar solution. Then the agar blocks were sliced to about 1 mm thickness. These slices were dehydrated with a graded ethanol series and embedded in LR White resin (London Resin, Hampshire, UK). Ultrathin sections (200 nm) were prepared with a diamond knife using a Leica Ultracut UCT (Leica, Wetzlar, Germany). Ultrathin sections were treated with blocking solution of 2 % (w/v) goat serum albumin in 0.1 M sodium phosphate buffer (pH 7.2) for 1 h at room temperature. The blocked sections were then incubated with anti-GFP antibody (1:500; Covance, Princeton, NJ, USA) for 1 h at room temperature. After washing with 0.1 M sodium phosphate buffer (pH 7.2), the sections were incubated with a solution of 10 nm gold-labeled goat anti-rabbit IgG antibody (1:50; Abcom, Cambridge, UK) in the blocking solution for 1 h at room temperature. Then the reacted sections were stained with 2 % (w/v) uranyl acetate. The stained sections were examined under transmission electron microscopy (JEM-1220; JEM, Tokyo, Japan) at 80 kV.

## Results

### Accumulation of prolamin–GFP fusion protein in transgenic rice seed

**Fig. 2:**
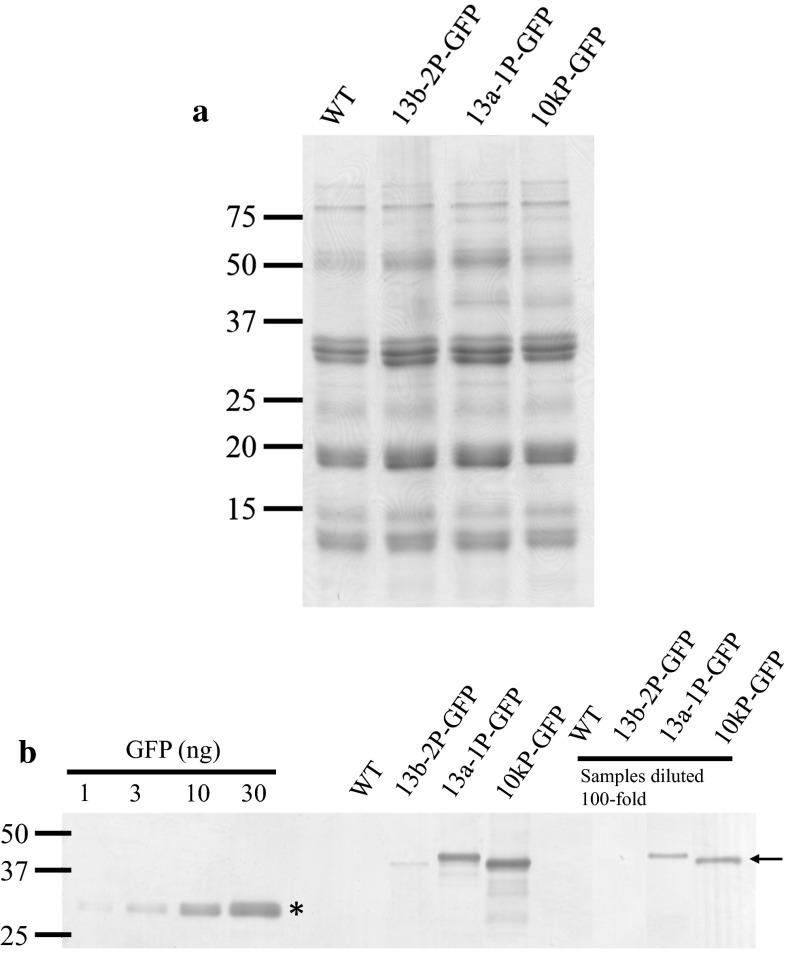
Accumulation of prolamin–GFP fusion protein in transgenic rice seed. **a** 10 μg of total proteins from each transgenic rice seed of WT, 13b-2P-GFP, 13a-1P-GFP, and 10kP-GFP was separated by SDS-PAGE. Arrowheads indicate the bands of prolamin–GFP fusion proteins. **b** Accumulation of rolamin–GFP fusion proteins was analyzed by immunoblot using anti-GFP antibody. The *lower bands* (*asterisk*) are recombinant GFP standards (1, 3, 10, 30 ng per *lane*) for quantitation. The *upper bands* (*arrows*) correspond to prolamin–GFP fusion proteins of 13b-2P-GFP, 13a-1P-GFP, and 10kP-GFP. Each sample is loaded with 10 μg or 100 ng of total proteins (diluted samples by 100-fold) for analyzing the bands without saturating intensities of the bands

Prolamin–GFP fusion proteins were detected as bands with molecular sizes of approximately 40 kDa in 13a-1P-GFP and 10kP-GFP, but not detected in 13b-2P-GFP with CBB staining (Fig. 2a, arrowheads). Prolamin–GFP fusion proteins were therefore detected by immunoblotting using anti-GFP antibody. The standard curves were made with 1, 3, 10, and 30 ng of recombinant GFP. The concentration of prolamins–GFP fusion proteins in 13b-2P-GFP, 13a-1P-GFP, and 10kP-GFP transgenic rice seeds were 0.09, 0.46, and 2.41 % of total protein contents, respectively (Fig. 2b).

### Localization of prolamin–GFP fusion proteins in endosperm tissue of transgenic rice seed

**Fig. 3:**
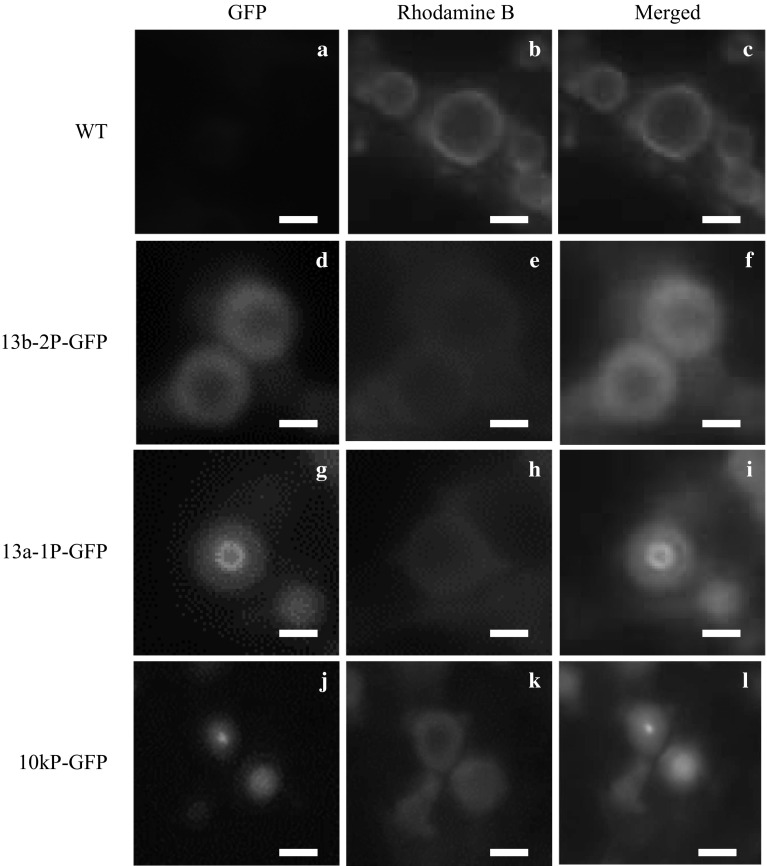
Localization of GFP in endosperm tissue of transgenic rice seed. The fluorescence images of WT (**a**–**c**), 13b-2P-GFP (**d**–**f**), 13a-1P-GFP (**g**–**i**), and 10kP-GFP (**j**–**l**) mature seeds were captured by fluorescence microscopy. The images in *green* (**a**, **d**, **g**, **j**) indicate GFP fluorescent signals. The images in *red* (**b**, **e**, **h**, **k**) indicate the fluorescence of rhodamine B, which is the pigment staining the peripheral region of PB-Is. The merged images are **c**, **f**, **i**, and **l**. *Bars* 1 μm (colour figure online)

The organelle localization of GFP in endosperm cells was investigated using fluorescence microscopic analysis. Green indicates GFP, and red indicates fluorescence of rhodamine B that stains the peripheral region of PB-I (Fig. 3). So GFP localization in specific layers of PB-Is could be observed according to the staining region of rhodamine B (Choi et al. 2000). The wild type has no GFP fluorescence, and the peripheral region of PB-I was stained with rhodamine B (Fig. 3, WT). In the 13b-2P-GFP line, the GFP signal seems to be located in the outer-most layer, where the signal overlaps with the rhodamine B (Fig. 3, 13b-2P-GFP), indicating that prolamin–GFP fusion proteins were localized in the outer-most layer of PB-Is. In the 13a-1P-GFP line, a GFP signal was observed as a ring shape inside the signal of rhodamine B (Fig. 3, 13a-1P-GFP), indicating that prolamin–GFP fusion proteins were localized in the middle layer of PB-Is. In the 10kP-GFP line, GFP was detected in the core region of PB-Is (Fig. 3, 10kP-GFP), indicating that prolamin–GFP fusion proteins were localized in the core of PB-Is. This localization of prolamin–GFP fusion proteins coincided with the positions of native prolamin species in the PB-I (Saito et al. 2012).

### In vitro pepsin digestion of transgenic rice seeds

**Fig. 4:**
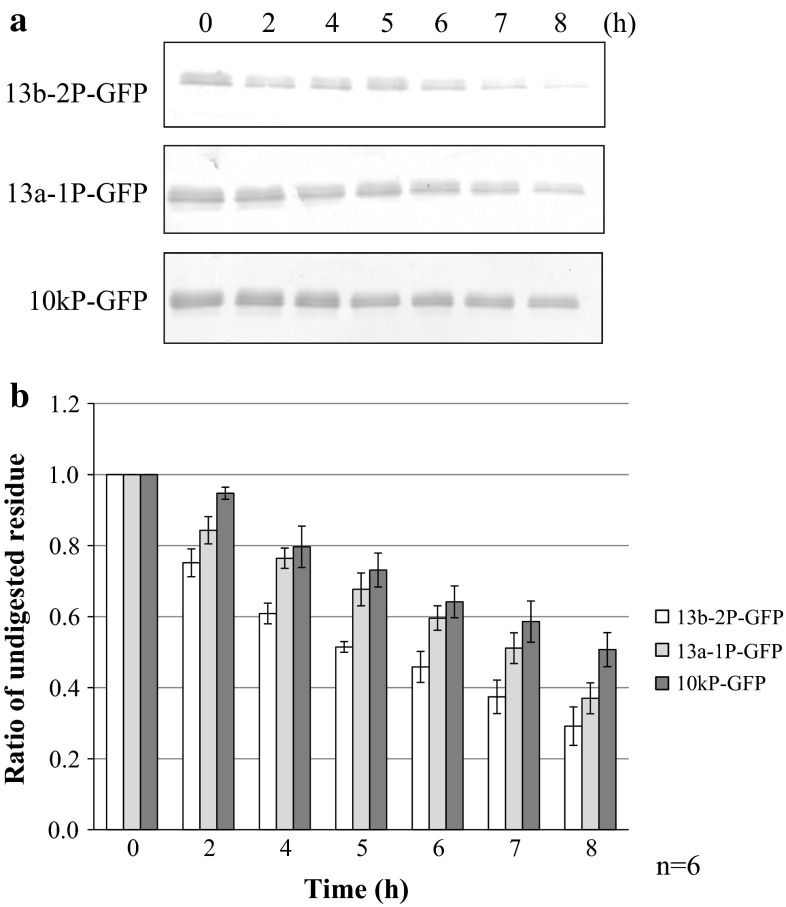
In vitro pepsin digestibility of prolamin–GFP fusion proteins in transgenic rice seeds of 13b-2P-GFP, 13a-1P-GFP, and 10kP-GFP. **a** Undigested prolamin–GFP fusion proteins in 13b-2P-GFP, 13a-1P-GFP, and 10kP-GFP were analyzed by immunoblot using anti-GFP antibody. **b** Ratios of undigested residue of prolamin–GFP fusion proteins at 0, 2, 4, 5, 6, 7, and 8 h. Data are mean ± SE of six independent replicates

The resistance against gastric protease of prolamin–GFP fusion proteins in PB-Is was confirmed by an in vitro pepsin digestion experiment. In the results of immunoblot analysis, prolamin–GFP fusion proteins seemed to be slightly decreased after degradation in the digested pellets of three transgenic rice seed samples (Fig. 4a). Then, these bands were quantified and the ratios of undigested residue were compared among the three lines. The histogram shows that prolamin–GFP fusion proteins in the 13b-2P-GFP line were digested faster than those in the 13a-1P-GFP line and more slowly than those in the 10kP-GFP line (Fig. 4b).

### Observation of PB-Is accumulating prolamin–GFP fusion proteins using transmission electron microscopy

**Fig. 5:**
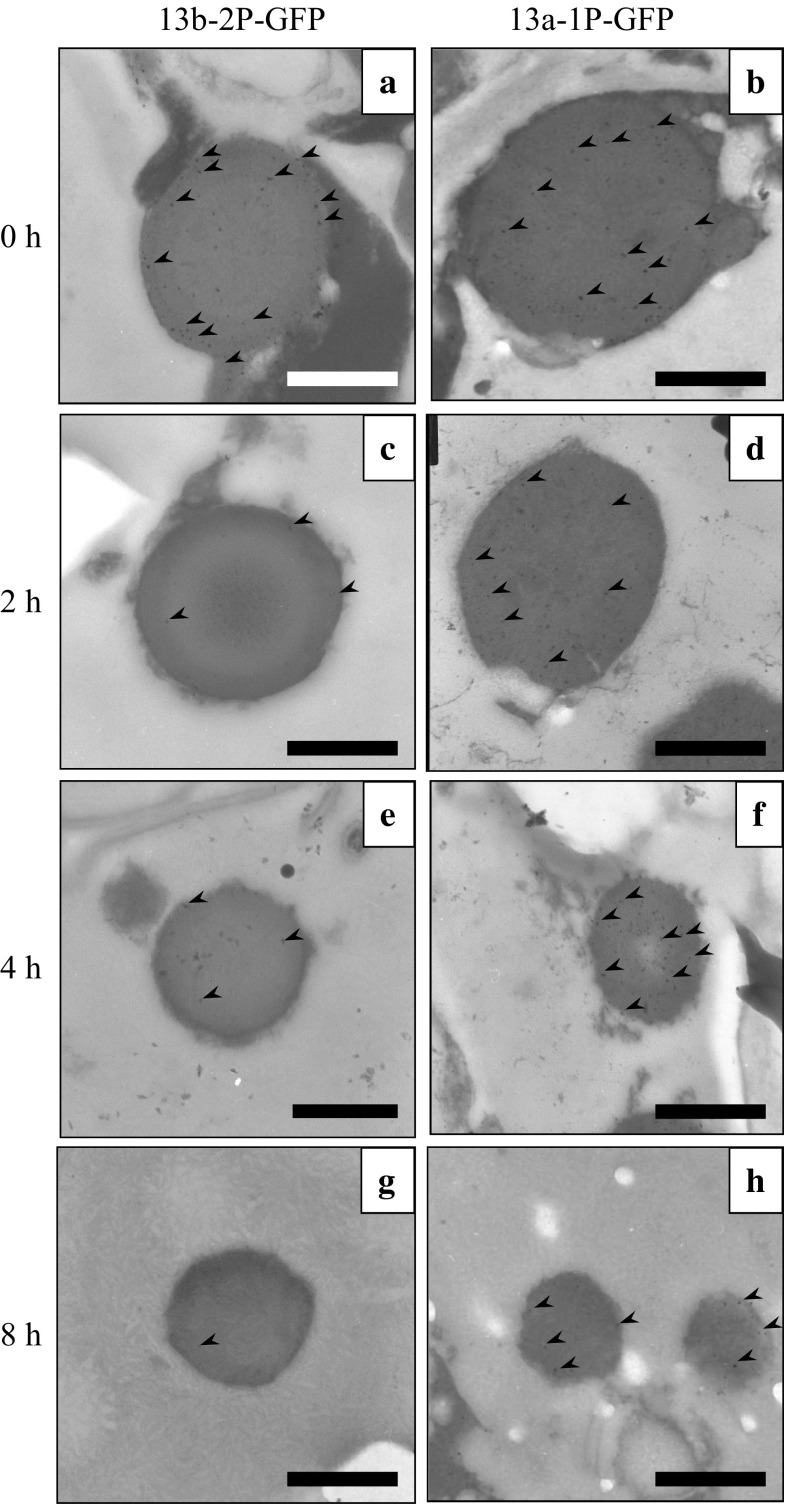
Immuno-transmission electron microscopy of the PB-Is after in vitro pepsin digestion. The pellets after in vitro pepsin digestion in 13b-2P-GFP (**a**, **c**, **e**, **g**) and 13b-1P-GFP (**b**, **d**, **f**, **h**) were subjected to morphologic observation of the PB-Is. The samples digested at 0 (**a**, **b**), 2 (**c**, **d**), 4 (**e**, **f**), and 8 (**g**, **h**) h were observed by microscopic analysis using anti-GFP antibody. *Arrowheads* indicate gold particles. *Bars* 1 μm

The morphology of PB-I was observed by immuno-transmission electron microscopic analysis of the pellets after pepsin digestive reaction (Fig. 5). The digested samples of 13b-2P-GFP and 13a-1P-GFP were compared at 0, 2, 4, and 8 h treatment. In both of these transformants, the diameters of PB-Is were measured at about 2 μm at 0 h and gradually decreased, reaching 1 μm at 8 h. These results imply that PB-Is are digested slowly from the peripheral region. In the samples at the 0 h, GFP antibodies reacted with outer-most layer in the 13b-2P-GFP, and reacted with middle layer of PB-Is in the 13a-1P-GFP (Fig. 5a, b). These results supported fluorescence microscopic observation (Fig. 3). In the samples that underwent the 8 h digestive reaction, GFP antibodies reacted near the peripheral region of spherical structures, whose diameters were approximately 1 μm in 13a-1P-GFP (Fig. 5h). On the other hand, there is only a few GFP antibody reacted with the spherical structures in 13b-2P-GFP (Fig. 5g). Thus, these spherical structures digested by the pepsin were PB-I. This result suggests that a portion of prolamin–GFP fusion proteins that accumulated in PB-Is were exposed on the outside.

## Discussion

In this study, we generated transgenic rice prolamin–GFP fusion proteins using a native promoter for prolamins and found it possible to control the localization of foreign proteins artificially in specific layers in PB-I. Furthermore, PB-Is possess resistance against gastric acid and pepsin. In fact, PB-Is were gradually digested from the outer-most side by pepsin treatment for long periods.

The accumulation ratios of prolamin–GFP fusion proteins in 13b-2P-GFP, 13a-1P-GFP, and 10kP-GFP transgenic rice seeds were 0.09, 0.46, and 2.41 % of total protein, respectively. These results showed that the accumulation ratio differed according to the transgenes of each prolamin. Qu and Takaiwa (2004) reported the expression strengths of several specific promoters in rice endosperm tissues. Using a GUS reporter assay, they showed that the promoter activity of 10 kDa prolamin was five times that of the 13 kDa prolamin. So, the differences in the GFP accumulation ratio of total proteins among the 10kP-GFP, 13a-1P-GFP, and 13b-2P-GFP rice seeds were a result of the expression strength differences among the prolamin promoters. In the case of the 13 kDa prolamin, Saito et al. (2012) reported that 13a prolamin contains six genes. On the other hand, 13b prolamin contains 22 genes. These results suggest that the expression activity of each 13b prolamin promoter is weak. However, 13b prolamin polypeptides were translated by a number of 13b prolamin genes in endosperm cells at the developing stage of rice seeds. As a result, 13b prolamin polypeptides could accumulate in large amounts.

Prolamin–GFP fusion proteins expressed by each native prolamin promoter were localized in the same region of each native prolamin. Our results suggested that the stage-specific expression of prolamin gene dependent on its promoter sequence is the cause of the specific localization of them. Saito et al. (2009) demonstrated that the 13a prolamin–GFP fusion proteins expressed by the 35S CaMV promoter was localized in the core region of PB-Is in transgenic rice seeds. In contrast, GFP expressed by the 35S CaMV promoter was detected in cytosol. These results suggested that GFP expressed as a fusion protein with 13a prolamin was targeted within the core of PB-Is, but that the location of 13a prolamin–GFP fusion proteins did not correspond to the localization of native 13a prolamins within PB-Is. Shigemitsu et al. (2013) reported that full prolamin polypeptide–GFP fusion proteins formed in ER derived protein bodies in transgenic rice calli. But the prolamin signal peptide–GFP fusion proteins was secreted into the intercellular space in transgenic rice calli. They demonstrated that mature polypeptides of prolamin possess the ability to retain in ER of heterologous transgene expression system. Similarly, our results indicated that mature polypeptides of each prolamin fused GFP were important to target the GFP in PB-Is of transgenic rice seeds rather than to control localization of GFP in specific layer of PB-Is. Furthermore, Saito et al. (2012) demonstrated that 13a prolamin–GFP fusion proteins and 10 kDa prolamin–GFP fusion proteins expressed by using each of the native prolamin promoters (*13a*-*1*_*pro*_*::13a*-*1:GFP* and *10* *kDa*_*pro*_*::10*  *kDa:GFP*) were localized in the middle layer of PB-Is and in the core of PB-Is, respectively. When the native promoter was used, the localization of each prolamin–GFP fusion protein coincided with the location of each native prolamin of the wild type. Saito et al. (2009, 2012) demonstrated that temporal control of each prolamin promoter is responsible for the localization of prolamin–GFP fusion protein within PB-I. But the analysis of transgenic rice seeds that expressed 13b-2 prolamin–GFP fusion proteins under the control of native promoter, was remained to be accomplished. In this study, transgenic rice expressing the 13b-2 prolamin–GFP fusion proteins under the control of the native promoter (13b-2P-GFP) was generated and the localization of 13b-2 prolamin–GFP fusion proteins was confirmed. As a result, 13b-2P–GFP fusion proteins were localized in the outer-most layer of PB-I. This result, together with the results for 13a-1P-GFP and 10kP-GFP, suggested that the localization of prolamin–GFP fusion proteins can be controlled in the outer-most layer, in the middle layer, or in the core of PB-Is by using each native prolamin promoter.

Some reports indicated that PB-I was hard to digest against pepsin treatment in vitro or protease in the rat gastrointestinal tract (Kumagai et al. 2006; Kubota et al. 2010, 2014). Therefore, we investigated the remaining protein contents when we performed in vitro pepsin digestion. Almost all fusion proteins remained at 8 h after pepsin digestion, but the digestion rate of fusion proteins against pepsin was different in each transgenic line. The prolamin–GFP fusion proteins decreased first in 13b-2P-GFP, then in 13a-1P-GFP, and finally in 10kP-GFP. Thus, the results of pepsin treatment suggested that PB-Is were digested slowly from the outer-most layer in the human stomach. Immuno-transmission electron microscopic analysis shows that PB-Is in 13b-2P-GFP or 13a-1P-GFP transgenic rice seed became gradually smaller, while the surfaces of the PB-Is became rougher, when the samples were treated by pepsin for 2, 4, and 8 h. A combination of immunoblot analysis and immuno-transmission electron microscopic analysis suggested that prolamin–GFP fusion proteins were exposed on the outside. Kubota et al. (2014) showed that the differences of prolamin digestibility between raw rice and cooked rice by feeding of mice. They reported that prolamin in raw rice was gradually digested through the alimentary tract of mice, while prolamin of cooked rice became indigestible. These results on the digestibility of PB-Is could be quite important for the development of transgenic rice seeds that accumulate useful proteins in PB-Is and can be used as medicine. There are some reports about the development of transgenic rice seed for pharmaceutical utilization. For example, Nochi et al. (2007) generated transgenic rice expressing a large amount of CTB and examined whether it was feasible as an edible vaccine by milling the rice into flour and feeding it to mice. They generated transgenic rice that expressed CTB fused with a KDEL signal used as an ER retention signal under the control of GluB promoter. In that case, CTB was localized in PB-I and PB-II, and it is not sufficient to accumulate CTB within PB-Is only using the KEDL signal. Our group also studied the utilization of PB-Is in rice seeds for oral vaccines by genetic engineering. In the present study, the localization of foreign proteins in certain layers of a PB-I was controlled in a case where foreign polypeptides are expressed as fusion proteins with each prolamin polypeptide under the control of each prolamin promoter. Takagi et al. (2010) tried to engineer the capsulation of the synthetic epitope 3Crp of Japanese cedar pollen allergen into PB-Is or PB-IIs of transgenic rice seeds. 3Crp peptides accumulated in PB-Is and remained for several hours, whereas 3Crp accumulating in PB-II dissolved in less than 2 min by pepsin or pancreatin in vitro. Furthermore, 3Crp accumulating in PB-Is more efficiently induced the immune tolerance of cedar pollen allergy than that in PB-IIs by feeding to mice. Thus, expressing vaccine antigen in PB-Is provides a low dose of administration that is sufficient to induce an immune response effectively. Kurokawa et al. (2014) analyzed MucoRice-CTB-RNAi seeds expressing CTB with an RNAi cassette of 13 kDa prolamin and glutelin A. MucoRice-CTB-RNAi seeds accumulated more CTB than transgenic rice seeds without the RNAi cassette, while 13 kDa prolamins and glutelins decreased. Kurokawa et al. (2014) showed that CTB in MucoRice-CTB-RNAi seeds was localized in PB-I, PB-II, plasma membrane, cell wall, and PB-like structure. The CTB accumulation level increased in MucoRice-CTB-RNAi seeds, but the population of PB-Is decreased. Thus, it is necessary to confirm the efficiency of oral vaccines in cases where they are useful for expressing target proteins in PB-Is or in another organelle. In this study, we found it possible to control the localization of foreign proteins such as GFP in specific layers of PB-Is. If the same results are acquired in the case of other useful proteins such as vaccine antigens, this method has the potential to lead to a novel technique for developing effective carriers of oral vaccines.

### **Author contribution statement**

T.M. designed this research. T.S., Y.S., and S.M. conducted experiments. M.T. contributed to the improvement of the in vitro pepsin digestion experiment. A.S. performed experiments and wrote this manuscript. All authors read and approved the manuscript.

## Abbreviations

CaMV: Cauliflower mosaic virus
CTB: Cholera toxin B subunit
ER: Endoplasmic reticulum
PB-I: Protein body type I
PB-II: Protein body type II
SDS-PAGE: Sodium dodecyl sulfate–polyacrylamide gel electrophoresis
WT: Wild type
